# Serum OxLDL Levels Are Positively Associated with the Number of Ischemic Events and Damaged Blood Vessels in Patients with Coronary Artery Disease

**DOI:** 10.3390/healthcare13121426

**Published:** 2025-06-14

**Authors:** Mariana Perez-Robles, Wendy Campos-Perez, Sissi Godinez-Mora, Victor Lopez-Alvarez, Liliana Estefanía Ramos-Villalobos, Felisardo Corona-Ruiz, Juan Armendariz-Borunda, Erika Martinez-Lopez

**Affiliations:** 1 Instituto de Nutrigenética y Nutrigenómica Traslacional, Departamento de Biología Molecular y Genómica, Centro Universitario de Ciencias de la Salud, Universidad de Guadalajara, Guadalajara 44340, Jalisco, Mexico; mariana.perez@academicos.udg.mx (M.P.-R.); wendy.campos4381@academicos.udg.mx (W.C.-P.); sissi.godinez5582@alumnos.udg.mx (S.G.-M.); victor.lopez1906@alumnos.udg.mx (V.L.-A.); 2 Doctorado en Ciencias de la Nutrición Traslacional, Departamento de la Reproducción Humana, Crecimiento y Desarrollo Infantil, Centro Universitario de Ciencias de la Salud, Universidad de Guadalajara, Guadalajara 44340, Jalisco, Mexico; 3 Servicio de Cardiología del Hospital Civil de Guadalajara “Dr. Juan I. Menchaca”, Guadalajara 44340, Jalisco, Mexico; liliana.ramos5375@academicos.udg.mx; 4 Clínica Medicina Familiar 1 del ISSSTE “Dr. Arturo González Guzmán”, Guadalajara 44340, Jalisco, Mexico; fexfcr@gmail.com; 5 Instituto de Biología Molecular en Medicina y Terapia Génica, Centro Universitario de Ciencias de la Salud, Universidad de Guadalajara, Sierra Mojada 950, Independencia Oriente, Guadalajara 44340, Jalisco, Mexico; socorro.armendariz@academicos.udg.mx

**Keywords:** coronary artery disease, cardiovascular disease, oxidized LDL, oxidized HDL, ischemic events, damaged blood vessels

## Abstract

**Background/Objectives:** The severity of coronary artery disease (CAD) depends on several factors. Oxidative stress contributes to the oxidation of lipoproteins, thereby promoting ischemic events (IEs) and damaged blood vessels (DBVs). This study aimed to compare biochemical variables and levels of oxidized lipoproteins in CAD patients stratified by the number of IEs and DBVs. **Methods:** A cross-sectional study was performed, including 51 patients diagnosed with CAD. Serum oxidized lipoproteins were measured by ELISA. **Results:** CAD patients with ≥2 IEs and ≥2 DBVs showed higher oxLDL levels than those with one IE and one DBV (8293.35 ng/mL [7131.32–9287.39] vs. 6474.26 ng/mL [5374.52–7574.01], *p* = 0.029). OxLDL levels were positively associated with the number of IEs (r^2^ = 15.2, B = 0.142 [0.046–0.239], *p* = 0.005) and DBVs (r^2^ = 19.2, B = 0.196 [0.018–0.374], *p* = 0.020). **Conclusions:** This study identified a positive association between elevated serum oxLDL levels and the presence of ≥2 IEs and ≥2 DBVs in CAD patients.

## 1. Introduction

Coronary artery disease (CAD) refers to a group of disorders in which the myocardium receives insufficient blood flow and oxygen due to arterial obstruction, most commonly caused by atheroma [1]. CAD is the most prevalent cardiovascular disease, responsible for 17.9 million deaths worldwide each year, and is the leading cause of mortality [2,3].

Clinically, CAD may present as chronic heart disease (stable angina) or acute coronary syndrome, which includes ST-segment-elevation myocardial infarction (STEMI), non-ST-segment-elevation myocardial infarction (NSTEMI), unstable angina, and mixed CAD [4,5]. Regardless of the type, patients with CAD are commonly treated with pharmacological and surgical interventions; however, most remain at risk of experiencing a second ischemic event (IE), which may affect additional blood vessels [6].

The main risk factors for a second IE and damage to two or more blood vessels are related to the development of atherosclerosis. These include an age >45 years in men and >55 years in women, male sex, and the presence of metabolic conditions such as obesity, arterial hypertension, type 2 diabetes mellitus (T2DM), and dyslipidemias—including hypercholesterolemia, hypertriglyceridemia, elevated low-density lipoprotein cholesterol (LDL), and hypoalphalipoproteinemia [7,8].

In addition, oxidative stress plays an important role in CAD [9,10]. Specifically, peroxyl radicals, fatty hydroperoxides, epoxides, isoprostanes, and aldehydes modify the lipid components of lipoproteins—particularly their polyunsaturated fatty acids (PUFAs)—and alter the structure of cysteine, methionine, and tyrosine residues in apolipoproteins found in native LDL and HDL. This leads to the formation of oxidized low-density and high-density lipoproteins (oxLDLs and oxHDLs, respectively) in the intima of blood vessels, where they are phagocytosed by macrophages forming foam cells. These oxidized lipoproteins exhibit alterations in charge, activity, function, and metabolism, and also promote the secretion of adhesion molecules and proinflammatory cytokines. This process culminates in the formation of atherosclerotic plaques in the intima, which may erode and lead to thrombus formation, resulting in vascular occlusion, CAD progression, or even a new IE in patients already diagnosed with the disease [11,12,13].

Therefore, it has been proposed to evaluate biomarkers associated with oxidative stress in patients with CAD, such as oxLDL and oxHDL, and to analyze their relationship with the number of IEs and damaged blood vessels (DBVs), as this has not yet been explored in the Mexican population. The aim of this study was to compare biochemical variables, including oxidized lipoproteins, in CAD patients stratified by the number of IEs and DBVs.

## 2. Materials and Methods

### 2.1. Study Subjects

A total of 51 individuals from western Mexico with a confirmed diagnosis of CAD participated in this cross-sectional study. These participants were enrolled between 2022 and 2023 through two medical centers: the Cardiology Service of the “Hospital Civil de Guadalajara, Dr. Juan I. Menchaca”, and the Family Medicine Clinic “Instituto de Seguridad y Servicios Sociales de los Trabajadores del Estado (ISSSTE), Dr. Arturo González Guzmán.” The inclusion criteria were an age between 30 and 75 years, a body mass index (BMI) ≥ 18.5 kg/m^2^, and a confirmed CAD diagnosis. Patients with autoimmune diseases, cancer, or chronic kidney disease were excluded. In addition, individuals with incomplete data or insufficient biological samples were excluded. Study evaluations were conducted at the “Instituto de Nutrigenética y Nutrigenómica Traslacional” affiliated with the “Universidad de Guadalajara”.

### 2.2. CAD Diagnosis and Cardiological Evaluations

CAD diagnosis and the number of IEs were established by a cardiologist through the assessment of clinical symptoms of ischemia. In men, oppressive chest pain radiating to the left arm, retrosternal pain, and dyspnea at rest or during exertion were evaluated. In women, atypical symptoms without chest pain—such as extreme fatigue, cough, palpitations, dizziness, nausea, gastrointestinal discomfort, shortness of breath, and numbness in the arms, back, jaw, or neck—were considered. Biomarkers of myocardial damage were also considered, specifically, elevated serum cardiac enzymes: creatine kinase ≥12 IU/L, hs-TnI >34 ng/L and hs-TnT >22 ng/L for men, and hs-TnI >15 ng/L and hs-TnT >14 ng/L for women. Electrocardiography was used to assess ST-segment abnormalities. In addition, coronary angiography was performed by a cardiologist to confirm arterial obstruction and determine the number of DBVs [14].

CAD diagnosis was based on the guidelines of the American College of Cardiology (ACC), the American Heart Association (AHA), and the European Society of Cardiology. Patients were categorized as having either chronic CAD (e.g., stable angina) or acute forms (STEMI, NSTEMI, or unstable angina). The mixed CAD type was considered when patients did not meet the criteria for one of the previous diagnoses [4,5].

### 2.3. Blood Pressure Evaluation

Blood pressure was measured using an automated upper-arm digital device (HEM-7130, Omron Healthcare Co., Ltd., Kyoto, Japan), following the recommendations outlined in the ACC/AHA guidelines [15].

### 2.4. Pharmacological and Interventional Treatment

Pharmacological treatments were categorized by function into the following groups: anticoagulants and antiplatelets (acetylsalicylic acid, clopidogrel), vasodilators (isosorbide, nifedipine), antihypertensives (metoprolol, losartan, enalapril), lipid-lowering agents (atorvastatin, pravastatin, ciprofibrate, bezafibrate), and glucose-control medication (metformin). Additionally, coronary stent placement was considered an interventional treatment.

### 2.5. Biochemical Analysis

A peripheral blood sample was collected after a fasting period of 8–10 h. The samples were then centrifuged for 15 min at 3500 rpm (revolutions per minute) to separate the serum. Total cholesterol (TC), high-density lipoprotein cholesterol (HDL), and triglycerides (TG) were measured using dry chemistry with a Vitros 350 Analyzer (Ortho-Clinical Diagnostics, Johnson & Johnson Services Inc., Rochester, NY, USA). Low-density lipoprotein cholesterol (LDL) was calculated using the Friedewald formula when TG concentrations were <400 mg/dL [5]. In addition, the TG/HDL and TC/HDL ratios were calculated by dividing TG by HDL, and TC by HDL, respectively.

### 2.6. Metabolic Comorbidities and Dyslipidemias

T2DM and arterial hypertension diagnoses were established by an internist according to the guidelines of the American Diabetes Association (ADA) and the American College of Cardiology/American Heart Association (ACC/AHA), respectively [16,17]. Dyslipidemia classifications were based on the thresholds defined by the ACC/AHA. Individuals were categorized as having hypertriglyceridemia when TG levels were ≥150 mg/dL, hypercholesterolemia when TC was ≥ 200 mg/dL, and elevated LDL-C when these levels were ≥70 mg/dL. Low HDL (hypoalphalipoproteinemia) was defined as levels <50 mg/dL for women and <40 mg/dL for men [17].

Thresholds for elevated lipid ratios were defined as a TG/HDL ratio >3.75 in men and >3.0 in women, and a TC/HDL ratio >5.0 in men and >4.0 in women [18,19].

### 2.7. Quantification of Serum oxLDL and oxHDL

Serum levels of oxLDL and oxHDL were measured using a sandwich-type enzyme-linked immunosorbent assay (ELISA), following the manufacturer’s protocol (Cell Biolabs, Inc., San Diego, CA, USA). Quantification was performed using a ThermoFisher Scientific Multiskan SkyHigh Microplate spectrophotometer (Waltham, MA, USA) at a wavelength of 450 nm. oxLDL/LDL and oxHDL/HDL ratios were calculated converting oxLDL and oxHDL values to mg/dL, and dividing oxLDL by LDL and oxHDL by HDL, respectively. For data analysis, ratio values were log-transformed using the natural logarithm (ln).

### 2.8. Anthropometric Measurements

Anthropometric measurements were taken after an 8-10 h fast, with participants barefoot and wearing light clothing. Body weight was measured with a SECA 803 scale, while height was measured using an SECA stadiometer (GMBH & Co., Hamburg, Germany). Waist circumference (WC) was assessed using a Lufkin Executive^®^ Thinline measuring tape (2 mm, New Brighton, MN, USA). Body fat percentage (BF%) was determined using tetrapolar electrical bioimpedance (Quantum V, RJL Systems, Clinton Township, MI, USA). The BMI was calculated as the weight in kilograms divided by the height squared in meters (kg/m^2^).

A waist circumference ≥88 cm in women and ≥102 cm in men was considered indicative of abdominal obesity [20]. The BF% was considered to be excessive when it was ≥25% in men and ≥35% in women [21]. A BMI of 25–29.9 kg/m^2^ was classified as overweight, and ≥30 kg/m^2^ as obesity [22].

### 2.9. Alcohol Consumption

Alcohol intake was assessed through patient self-report, including details on the volume, frequency, and duration of consumption. Significant alcohol intake was defined as >14 g/day for women and >28 g/day for men over the past year, or consumption of ≥5 alcoholic drinks for men or ≥4 for women on the same occasion on at least one day in the past month [23,24]. Additionally, the Alcohol Use Disorders Identification Test (AUDIT) was administered to evaluate risky alcohol intake. A score ≥8 in men and ≥5 in women was considered indicative of risk [25].

### 2.10. Tobacco Index and Risk Exposure

The tobacco index was determined according to pack-years, obtained by multiplying the daily number of cigarettes by the total number of years of smoking, and dividing the result by 20. The cut-off values were defined as follows: low (<5), moderate (5–15), and high (>15) [26]. Tobacco exposure risk was also assessed using the Tobacco Scale for Primary Care (Escala de Tabaquismo en Atención Primaria, ETAP). A value ≥ 20 years on the ETAP was considered indicative of high-risk exposure [27].

### 2.11. Statistical Analysis

The sample size was calculated using a formula for studies assessing the correlation between two quantitative variables, based on data obtained from a study conducted in Augsburg that evaluated the relationship between serum oxLDL levels and the risk of an IE [28]. A significance level of *p* = 0.05, a statistical power of 80%, and an estimated 20% attrition rate were considered, resulting in a required sample of 44 participants.

The Shapiro–Wilk test was used to assess the normal distribution of variables. Descriptive statistics for qualitative variables were reported as frequencies and percentages. Comparative analyses of quantitative variables, adjusted for covariates (age, sex, alcohol consumption, smoking, and pharmacological treatment), were performed using a general univariate model. To analyze the association between two quantitative variables and to determine the correlation coefficient, a linear regression model was used. To determine the risk, binary logistic regression models were used. A 95% confidence interval and a *p* value <0.05 were considered statistically significant. Statistical analyses were performed using SPSS version 20.0 software (IBM Corp., Armonk, NY, USA).

## 3. Results

### 3.1. Characteristics of the Study Subjects

**Figure 1:**
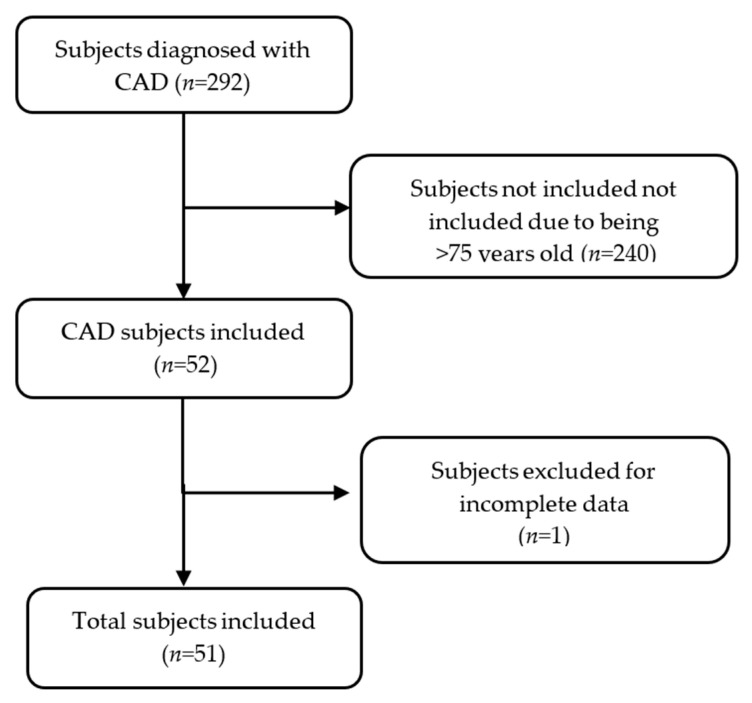
Flow diagram of study subjects.

In this study, 51 patients with CAD were included (Figure 1). Of the study participants, 67% (*n* = 34) were men and 33% (*n* = 17) were women, with a median age of 60 years (IQR: 55–68).

For clinical variables, the median systolic blood pressure (SBP) was 129 mmHg (IQR: 111–140), and the median diastolic blood pressure (DBP) was 78 mmHg (IQR: 71–86). Regarding cardiological variables, the median number of DBVs was 2 (IQR: 1–2), and the median number of IEs was also 2 (IQR: 1–2). For anthropometric variables, the median body mass index (BMI) was 28.4 kg/m^2^ (IQR: 26.6–30.9).

The numerical values of the remaining clinical and anthropometric variables are presented in Table 1.

With respect to biochemical variables, the average oxLDL levels were 6500 ng/mL (IQR: 5560–8320). The average oxHDL levels were 3.2 ng/mL (IQR: 2.7–3.6) in men and 3.4 ng/mL (IQR: 3.3- 4.3) in women. The oxLDL/LDL and oxHDL/HDL ratios are presented in Table 2.

### 3.2. Cardiological, Anthropometric, and Metabolic Variables

Regarding the types of CAD, 66.6% (*n* = 34) of patients presented with mixed CAD, 15.7% (*n* = 8) had STEMI, 15.7% (*n* = 8) had stable angina, and 2% (*n* = 1) had NSTEMI. Additionally, 51% (*n* = 26) of patients had two or more DBVs; likewise, 51% (*n* = 26) had two or more IEs. Based on this distribution, patients were categorized into two groups: those with one DBV and one IE, and those with more than two DBVs and more than two IEs. This grouping allowed for the exploration of whether these variables reflected increased clinical severity.

Regarding qualitative anthropometric analysis, 46% (*n* = 23) of patients were classified as overweight, and 40% (*n* = 20) as obese. In addition, 77.1% (*n* = 27) had an excessive body fat percentage (BF%), and 49% (*n* = 25) presented with abdominal obesity. The most common metabolic comorbidity was hypertension, present in 37 subjects (73%). Hypoalphalipoproteinemia was present in 70.6% of subjects (*n* = 36), hypertriglyceridemia in 53% (*n* = 27), and an elevated TC/HDL ratio in 62.7% (*n* = 32). Information on other comorbidities and lipid profile abnormalities is provided in Appendix A (Table A1). Additionally, data on alcohol and tobacco consumption are presented in Table A2.

### 3.3. Comparative Analysis in Subjects Classified by Cardiological Variables

Quantitative variables were compared among study subjects stratified by the number of ischemic events (IEs) and damaged blood vessels (DBVs), as shown in Table 3.

**Figure 2:**
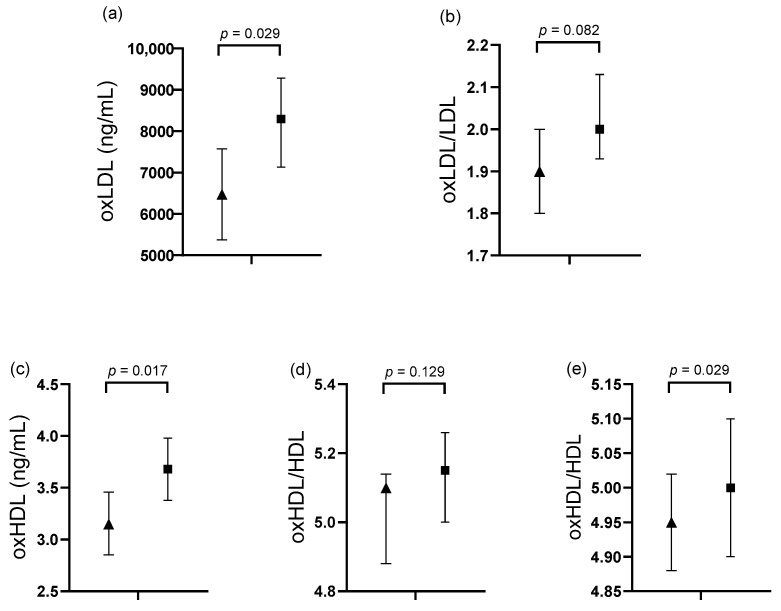
Comparative analysis of oxidized lipoproteins in subjects stratified by ischemic events (IEs) and damaged blood vessels (DBVs). ■ Subjects with 1 IE and 1 DBV. ▲ Subjects with ≥2 IEs and ≥2 DBVs. Panels show the following: (**a**) serum oxLDL levels, (**b**) oxLDL/LDL ratio, (**c**) oxHDL levels, and oxHDL/HDL ratio in (**d**) men and (**e**) women. Data are expressed as estimated means with 95% confidence intervals. *p* values were obtained using a general univariate model adjusted for age, sex, alcohol consumption, smoking, and pharmacological treatment. oxLDL/LDL and oxHDL/HDL ratios are reported as natural logarithms (ln). LDL: low-density lipoprotein cholesterol; HDL: high-density lipoprotein cholesterol; oxLDL: oxidized low-density lipoprotein; oxHDL: oxidized high-density lipoprotein. Units: oxLDL and oxHDL in ng/mL (nanograms per milliliter).

Subjects with ≥2 IEs and ≥2 DBVs had significantly higher oxLDL concentrations compared to those with 1 IE and 1 DBV (8293.35 ng/mL [7131.32–9287.39] vs. 6474.26 ng/mL [5374.52–7574.01], *p* = 0.029). Similarly, oxHDL levels were significantly higher in the ≥2 IEs and ≥2 DBVs group (3.68 ng/mL [3.38–3.98] vs. 3.15 ng/mL [2.85–3.46], *p* = 0.017). The oxHDL/HDL ratio was also significantly higher in the ≥2 IEs and ≥2 DBVs group compared to the other group (5.00 [4.9–5.1] vs. 4.95 [4.88–5.02], *p* = 0.029). A trend toward higher oxLDL/LDL ratios was observed in the ≥2 IEs and ≥2 DBVs group (*p* = 0.082) (Figure 2).

### 3.4. Association of oxLDL Levels with Cardiological Variables

A multivariable linear regression analysis was performed to assess the association between serum oxLDL levels and cardiological variables. All biochemical variables that showed a trend or statistical significance in Figure 2 were included in the multivariable model. A positive association was observed between serum oxLDL levels and the number of IEs (*R*^2^ = 0.152, *p* = 0.005) and DBVs (*R*^2^ = 0.192, *p* = 0.020), respectively (Table 4).

Additionally, an odds ratio (OR) analysis was performed, using the population mean serum oxLDL value (7358.82 ng/mL) as the cut-off point. An oxLDL value ≥7358.82 ng/mL was significantly associated with an increased risk of presenting with ≥2 IEs and ≥2 DBVs (OR = 4.667, 95% CI: 1.341–16.239, *p* = 0.012).

## 4. Discussion

It is well established that both genetic and environmental factors contribute to the development of chronic diseases, including coronary artery disease (CAD), which remains the most prevalent chronic condition and the leading cause of mortality in Mexico [29]. Although multiple studies have identified diverse risk factors for CAD, limited data are available on the recurrence and severity of ischemic events (IEs) in patients with existing cardiovascular damage [29]. Accordingly, this study aimed to analyze the clinical characteristics and risk factors associated with CAD development and recurrence in a Mexican population.

In terms of sex distribution, a higher percentage of male participants was observed, consistent with prior studies indicating that CAD disproportionately affects men. A descriptive study conducted in Mexico City involving 586 CAD patients reported that 70% were male [30]. This sex disparity is often attributed to hormonal factors—particularly estrogens—which promote vasodilation, reduce fibrosis, enhance mitochondrial function, and mitigate oxidative stress. As a result, premenopausal women exhibit lower susceptibility to CAD [31,32].

Mixed CAD was the most frequent subtype in this study, likely reflecting the coexistence of both stable and unstable atherosclerotic plaques [33]. This finding aligns with data from the 2016 National Registry of Acute Coronary Syndrome, which reported a 50% prevalence of mixed CAD [34]. However, updated prevalence data for CAD subtypes in the Mexican population are lacking.

Comorbidity analysis revealed a high prevalence of hypertension and type 2 diabetes mellitus (T2DM), both of which are strongly implicated in CAD pathogenesis through mechanisms such as endothelial dysfunction, oxidative stress, and chronic inflammation [35,36]. According to the 2023 ENSANUT survey, the prevalence of T2DM and hypertension prevalence in the general Mexican population was 18.4% and 29.9%, respectively [37]—notably lower than the rates observed in this study’s cohort, which likely reflects its clinical profile. These findings reinforce the contribution of these comorbidities as major risk factors for CAD progression.

Despite ongoing pharmacological treatment (Table A3), hypoalphalipoproteinemia and hypertriglyceridemia were highly prevalent. This may be partially attributable to the frequent use of atorvastatin, a lipid-lowering agent that is effective in reducing LDL-C, but has a limited impact on triglycerides and HDL-C levels [38]. Nevertheless, atorvastatin exerts antioxidant effects by enhancing paraoxonase-1 (PON1) activity, which inhibits LDL oxidation and foam cell formation, reinforcing its relevance in secondary prevention [39]. However, pharmacological strategies should be accompanied by lifestyle interventions, including dietary adjustments targeting an omega-6-to-omega-3 PUFA ratio ≤ 5:1, supplementation with 1.8 g/day of marine omega-3s, and personalized physical activity plans [40,41].

Anthropometric indicators, including overweight, an excessive body fat percentage, and abdominal obesity, were also highly prevalent. These conditions are closely linked to CAD, particularly abdominal fat, which has been shown to impair endothelial function, increase oxidative stress, and promote systemic inflammation. In this context, Xue et al. (2021) identified a dose-dependent relationship between abdominal fat and cardiovascular risk, emphasizing its impact on lipid dysregulation and the pathogenesis of dyslipidemia [42]. These findings underscore the importance of monitoring anthropometric variables in clinical practice and cardiovascular risk assessments.

Beyond metabolic disorders, environmental risk factors such as alcohol and tobacco use were also relevant in this cohort. Approximately 25% of participants reported alcohol consumption, and 7.8% exhibited high-risk intake. Wood et al. suggest that even moderate alcohol intake may exacerbate CAD progression by elevating systemic inflammation [43], partly through increased homocysteine, oxLDL, and oxidative stress levels [44].

Moreover, 38.5% of participants were current moderate or heavy smokers—similar to the AHA-reported rate of 35% among high-risk cardiovascular patients [45]. Passive smoke exposure was also high, potentially compounding cardiovascular risk through mechanisms such as platelet activation and reduced oxygen delivery [46].

In addition to hypertriglyceridemia and hypoalphalipoproteinemia, circulating oxidized lipoproteins—namely oxLDL and oxHDL—may serve as novel biomarkers of ischemic heart disease. In this cohort, significantly higher levels of both oxLDL and oxHDL were observed in individuals with more than two IEs and more than two DBVs, compared to those with a single event.

This finding is consistent with a 2022 case–control study by Yazdandoust et al., which examined 572 Iranian patients with stable angina and reported significantly elevated oxHDL levels in patients compared to controls (3.15 [1.01–1.31] vs. 2.85 [0.62–1.06] ng/mL, *p* < 0.001) [47]. However, their study did not investigate associations between oxHDL levels and the number of DBVs.

Functionally, HDL exerts antioxidant and anti-inflammatory effects, primarily through paraoxonase-1 (PON1) activity, which protects LDL from oxidative modification. Once oxidized, however, HDL loses its protective properties and can become proinflammatory and pro-atherogenic. Oxidized HDL also exhibits impaired reverse cholesterol transport, a key mechanism in atherosclerosis prevention [48].

In comparison, oxLDL levels in our study differed from those reported in a U.S. cohort of 304 individuals, where the mean serum oxLDL concentrations were 31,900 ± 10,200 ng/mL in patients with prior IEs [49]. Similarly, Wang et al. (2020) found that CAD patients with oxLDL ≥139.6 ng/mL had a significantly increased risk of recurrent IEs at 90 days (HR: 1.57) and at one year (HR: 1.49) [9]. Variability in oxLDL concentrations across studies may be attributable to differences in detection methods and the biomarkers selected. In our study, oxLDL was quantified using malondialdehyde (MDA) as a marker of lipid peroxidation. Nonetheless, despite these methodological differences, evidence consistently supports a link between elevated oxLDL levels and CAD risk.

Our findings suggest that elevated oxHDL concentrations and the strong correlation between oxLDL levels, IEs, and DBVs support the inclusion of these oxidized lipoproteins in standard lipid panels. Notably, an oxLDL value ≥ 7358.82 µg/mL was associated with a 4.6-fold increased risk of presenting with more than two IEs and more than two DBVs. Thus, the routine measurement of oxLDL and oxHDL should be considered both in healthy individuals and in patients with established CAD, given their relevance to oxidative stress, lipid peroxidation, disease severity, and recurrence. Importantly, these biomarkers can be incorporated into standard biochemical evaluations.

Although individuals with a history of cancer, chronic kidney disease, or autoimmune disorders were excluded from this study, residual confounding may persist due to the absence of data on key cardiovascular risk factors, including family history, inflammatory markers such as high-sensitivity C-reactive protein, and detailed measures of renal function. The omission of these variables from the regression models is a limitation that should be considered when interpreting the results. Future studies should aim to incorporate these factors to enhance risk adjustment and minimize confounding.

Additionally, the relatively small sample size limited the statistical power of subgroup analyses, particularly those stratified by sex or clinical severity. Therefore, caution is warranted when interpreting these findings. Future research should include larger cohorts and evaluate other cardiac biomarkers, such as small dense LDL and indicators of endothelial function.

Longitudinal studies are also needed to monitor oxLDL levels over time and across diverse populations in order to further validate their clinical utility. Moreover, targeted nutrigenetic and pharmacological interventions should be explored as strategies to reduce oxLDL concentrations and prevent recurrent vascular damage in CAD patients.

## 5. Conclusions

This study demonstrated that oxLDL concentrations exceeding 7358.82 ng/mL are associated with a 4.6-fold increased likelihood of experiencing a second ischemic event (IE) and presenting with more than one damaged blood vessel (DBV) in patients with coronary artery disease (CAD). Given the significant association observed between circulating oxLDL levels and cardiological variables, oxLDL emerges as a promising clinical biomarker for identifying individuals at elevated cardiovascular risk. Its inclusion in standard biochemical assessments may support the early implementation of targeted medical and nutritional interventions to reduce disease progression and recurrence.

## Figures and Tables

**Table 1:** Clinical and anthropometric characteristics of the study subjects (*n* = 51).

Variables	Values
Clinical	
SBP (mmHg)	129 (111–140)
DBP (mmHg)	78 (71–86)
DBVs (number)	2 (1–2)
IEs (number)	2 (1–2)
Anthropometric	
Body weight (kg)	74 (64.7–88)
BMI (kg/m^2^)	28.4 (26.6–30.9)
Body fat (%)	
M (*n* = 34)	28.1 (20.5–32.5)
W (*n* = 17)	42.1 (37.9–47.25)
WC (cm)	
M (*n* = 34)	98 (90–107)
W (*n* = 17)	97.5 (93.2–102.7)

Data are reported as median (25th–75th percentile). SBP: systolic blood pressure; DBP: diastolic blood pressure; DBVs: damaged blood vessels; IEs: ischemic events; BMI: body mass index; M: men; W: women; WC: waist circumference. Units: millimeters of mercury (mmHg); kilograms (kg); kilograms per square meter (kg/m^2^).

**Table 2:** Biochemical characteristics of the study subjects (*n* = 51).

Biochemical Variables	Values
TC (mg/dL)	139 (109–171)
LDL (mg/dL)	72 (45–98)
VLDL (mg/dL)	30 (21–41)
HDL (mg/dL)	
M (*n* = 34)	33 (25.5–40)
W (*n* = 17)	43 (33.5–51.5)
TG (mg/dL)	157 (104–208)
TG/HDL-c ratio	
M (*n* = 34)	4.67 (2.7–7.1)
W (*n* = 17)	3.94 (2.4–6.2)
TC/HDL-c ratio	
M (*n* = 34)	4 (3–5)
W (*n* = 17)	4 (2–5)
oxLDL (ng/mL)	6500 (5560–8320)
oxHDL (ng/mL)	
M (*n* = 34)	3.2 (2.7–3.6)
W (*n* = 17)	3.4 (3.3–4.3)
oxLDL/LDL	5.04 (4.9–5.12)
oxHDL/HDL	
M (*n* = 34)	5.04 (4.98–5.19)
W (*n* = 17)	5.03 (4.87–5.09)

Data are presented as median (25th–75th percentile). oxLDL/LDL and oxHDL/HDL ratios are reported as natural logarithms (ln). M: men; W: women; WC: waist circumference; TC: total cholesterol; LDL: low-density lipoprotein cholesterol; HDL: high-density lipoprotein cholesterol; oxLDL: oxidized low-density lipoprotein; oxHDL: oxidized high-density lipoprotein; TG: triglycerides. Units: TC, LDL, VLDL, HDL, and TG in mg/dL (milligrams per deciliter); oxLDL and oxHDL in ng/mL (nanograms per milliliter).

**Table 3:** Comparative analysis of biochemical variables in subjects classified by the number of IEs and DBVs.

Variables	1 IE and 1 DBV (*n* = 25)	≥2 IEs and ≥2 DBVs (*n* = 26)	*p* Value
TC (mg/dL)	148.15 (132.73–163.56)	138.39 (123.27–153.51)	0.371
LDL (mg/dL)	78.87 (64.84–92.90)	70.08 (53.33–83.83)	0.376
VLDL (mg/dL)	33.80 (27.08–40.52)	32.14 (25.56–38.73)	0.727
HDL (mg/dL)			
M (*n* = 34)	34.03 (29.55–38.59)	32.83 (28.82–36.85)	0.679
W (*n* = 17)	49.81 (36–63.58)	39.69 (23.10–56.27)	0.333
TG (mg/dL)	187.88 (147.55–228.21)	159.34 (119.80–198.87)	0.318
TG/HDL ratio			
M (*n* = 34)	5.99 (4.03–7.95)	5.18 (3.44–6.92)	0.932
W (*n* = 17)	4.23 (2.65–5.81)	4.33 (2.43–6.24)	0.534
TC/HDL-c ratio			
M (*n* = 34)	4.20 (3.43–4.97)	4.25 (3.57–4.94)	0.416
W (*n* = 17)	3.52 (2.59–4.46)	4.10 (2.97–5.22)	0.914

Values are presented as estimated means with 95% confidence intervals. *p* values were obtained using a general univariate model adjusted for age, sex, alcohol consumption, smoking, and pharmacological treatment. *p* values <0.05 were considered statistically significant. IEs: ischemic events; DBVs: damaged blood vessels; M: men; W: women; WC: waist circumference; TC: total cholesterol; TG: triglycerides; LDL: low-density lipoprotein cholesterol; HDL: high-density lipoprotein cholesterol. Units: TC, LDL, VLDL, HDL, and TG in mg/dL (milligrams per deciliter).

**Table 4:** Association of oxLDL serum levels with cardiological variables.

Model	Dependent Variable	R^2^	B (95% CI)	*p* Value
1	Number of ischemic events	0.152	0.142 (0.046–0.239)	0.005
2	Number of damaged blood vessels	0.192	0.196 (0.018–0.374)	0.020

Note: Linear regression models adjusted for age, sex, alcohol consumption, smoking, and pharmacological treatment.

## Data Availability

The data presented in this study are available upon reasonable request from the corresponding author.

## Abbreviations

The following abbreviations are used in this manuscript:

ACC	American College of Cardiology
ADA	American Diabetes Association
AHA	American Heart Association
AUDIT	Alcohol Use Disorders Identification Test
BF%	body fat percentage
BMI	body mass index
CAD	coronary artery disease
DBV	damaged blood vessel
ELISA	enzyme-linked immunosorbent assay
ETAP	*Escala de Tabaquismo para Atención Primaria* (Tobacco Scale for Primary Care)
HDL	high-density lipoprotein
IE	ischemic event
LDL	low-density lipoprotein
MDA	malondialdehyde
NSTEMI	non-ST-segment-elevation myocardial infarction
oxLDL	oxidized low-density lipoprotein
oxHDL	oxidized high-density lipoprotein
PON1	paraoxonase 1
PUFAs	polyunsaturated fatty acids
STEMI	ST-segment-elevation myocardial infarction
T2DM	type 2 diabetes mellitus
TC	total cholesterol
TG	triglycerides
WC	waist circumference

## Appendix A

**Table A1:** Frequencies of metabolic comorbidities and dyslipidemias in the study subjects (*n* = 51).

Variables	Values %, (n)
Metabolic comorbidities	
Hypertension	73.0 (37)
T2DM	49.0 (25)
Dyslipidemia	
Hypoalphalipoproteinemia	70.6 (36)
Hypertriglyceridemia	53 (27)
Elevated LDL	51 (26)
Hypercholesterolemia	12 (6)
Lipid ratios	
Elevated TC/HDL	62.7 (32)
Elevated TG/HDL	45.1 (23)

Note: Data are expressed as percentages and absolute frequencies. T2DM: type 2 diabetes mellitus; LDL-c: low-density lipoprotein cholesterol; TG: triglycerides; TC: total cholesterol.

**Table A2:** Alcohol and tobacco consumption among the study subjects (*n* = 51).

Variables	Values %, (n)
Alcohol consumption	
Alcohol consumption	24.0 (11)
High-risk alcohol consumption	7.8 (4)
Smoking	
Light smoking	23.0 (6)
Moderate smoking	38.5 (10)
Severe smoking	38.5 (10)
Suspended smoking	33.3 (17)
High-risk passive smoking	86.2 (25)

Note: Data are expressed as percentages and absolute frequencies.

**Table A3:** Pharmacological treatments among the study subjects (*n* = 51).

Medication Type	Medication	Values %, (n)
Anticoagulants and antiplatelets	Acetylsalicylic acid	60.8 (31)
	Clopidogrel	31.4 (16)
Vasodilators	Isosorbide	13.7 (7)
	Nifedipine	3.9 (2)
Antihypertensives	Metoprolol	47.1 (24)
	Losartan	29.4 (15)
	Enalapril	21.6 (11)
Lipid-lowering	Atorvastatin	58.8 (30)
	Bezafibrate	7.8 (4)
	Pravastatin	3.9 (2)
	Ciprofibrate	2 (1)
Glucose-control	Metformin	35.3 (18)

Note: Data are expressed as percentages and absolute frequencies.
